# Emerging Transcriptional Mechanisms in the Regulation of Epithelial to Mesenchymal Transition and Cellular Plasticity in the Kidney

**DOI:** 10.3390/jcm5010006

**Published:** 2016-01-12

**Authors:** Letizia De Chiara, John Crean

**Affiliations:** Diabetes Complications Research Centre, UCD School of Biomolecular and Biomedical Science, University College Dublin, Belfield, Dublin 4, Ireland; john.crean@ucd.ie

**Keywords:** EMT, Kidney, SNAI1, TGFβ signaling, plasticity, PRC2, SMAD3

## Abstract

Notwithstanding controversies over the role of epithelial to mesenchymal transition in the pathogenesis of renal disease, the last decade has witnessed a revolution in our understanding of the regulation of renal cell plasticity. Significant parallels undoubtedly exist between ontogenic processes and the initiation and propagation of damage in the diseased kidney as evidenced by the reactivation of developmental programmes of gene expression, in particular with respect to TGFβ superfamily signaling. Indeed, multiple signaling pathways converge on a complex transcriptional regulatory nexus that additionally involves epigenetic activator and repressor mechanisms and microRNA regulatory networks that control renal cell plasticity. It is becoming increasingly apparent that differentiated cells can acquire an undifferentiated state akin to “stemness” which is leading us towards new models of complex cell behaviors and interactions. Here we discuss the latest findings that delineate new and novel interactions between this transcriptional regulatory network and highlight a hitherto poorly recognized role for the Polycomb Repressive Complex (PRC2) in the regulation of renal cell plasticity. A comprehensive understanding of how external stimuli interact with the epigenetic control of gene expression, in normal and diseased contexts, establishes a new therapeutic paradigm to promote the resolution of renal injury and regression of fibrosis.

## 1. Introduction

**Figure 1:**
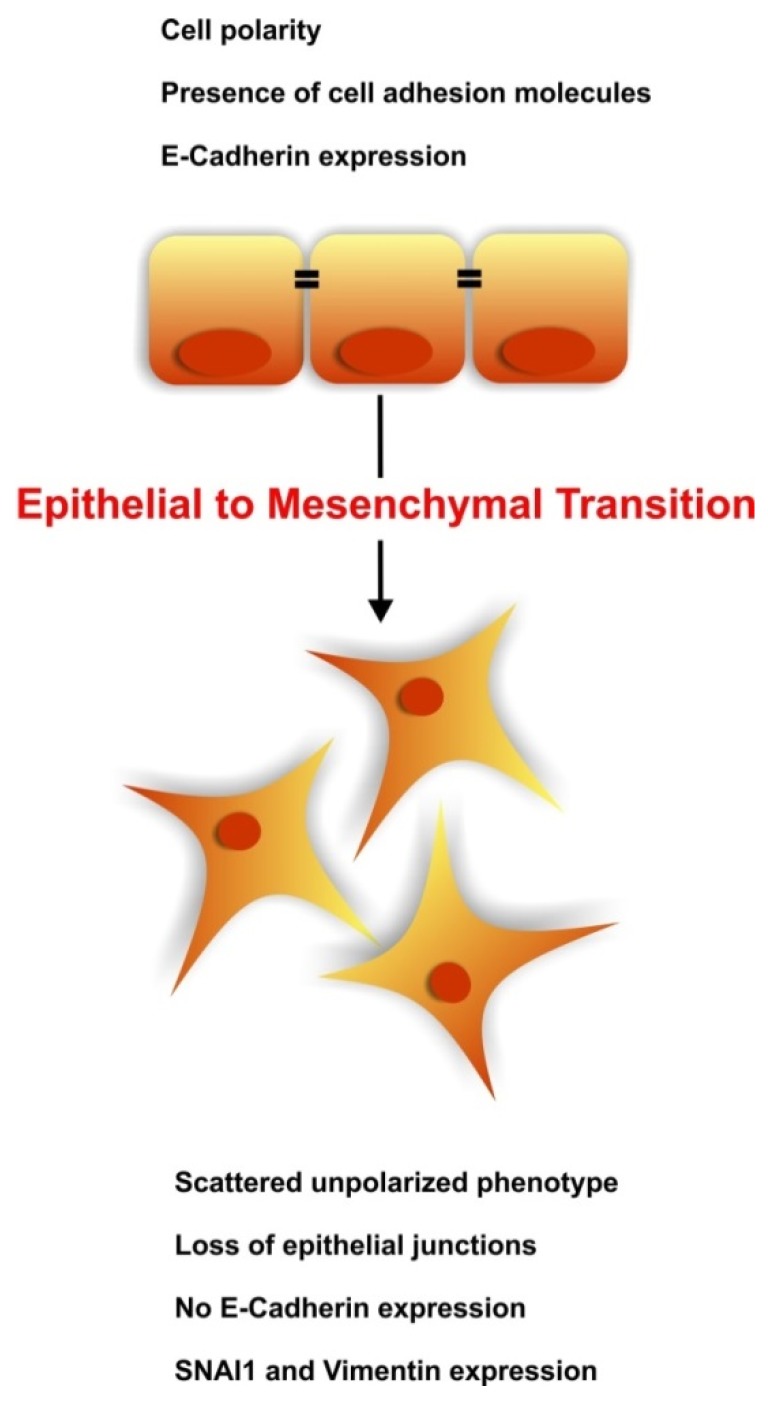
Epithelial to Mesenchymal transition. During Epithelial to Mesenchymal transition (EMT), epithelial cells lose their organized phenotype and gain a scattered mesenchymal phenotype. This transition can be caused by various growth factors and is characterized by loss of polarization, tight junctional integrity, and E-Cadherin expression. The epithelial cells undergoing EMT acquire a fibroblastic appearance, increased motility and *de novo* expression of SNAI1, Vimentin, α-SMA, and N-Cadherin.

Epithelial to Mesenchymal Transition (EMT) is a unique biologic process that involves distinct molecular reprogramming and phenotypic changes characterized by a transition from polarized epithelial cells to scattered mesenchymal cells, thus leading to increased motility and invasion (Figure 1). EMT is often regarded as a deleterious mechanism, leading to tumor invasion, metastasis, and fibrosis but is increasingly recognized as essential in multiple biological contexts [1]. EMTs are generally involved in three distinct biological processes that carry very different functional consequences. Type I EMT occurs during embryogenesis and leads to the formation of primary mesenchyma that will eventually turn into epithelia [2]; type II EMT is associated with wound healing, tissue regeneration, and organ fibrosis [3]; finally, type III EMT is typical of metastatic tumors [4]. Recently, EMT has been linked with the acquisition of pluripotency by somatic cells, namely induced Pluripotent Stem Cell (iPSC) formation [5]. Reprogramming of mouse embryonic fibroblasts (MEFs) into iPSCs by Shinya Yamanaka and colleagues has been recognized as a fundamental breakthrough in biology and medicine [6]. Since then, our understanding of the process has improved as the scientific community gained deeper insight into the mechanism of acquisition of pluripotency [7,8]. The required acquisition of a plastic phenotype is the thread that associates all these different EMT-processes. During these processes the cells adapt to micro-environmental stimuli by dynamic decisions that determine changes in microRNAs, transcription factors (TFs), and epigenetic modifiers of gene expression. This network exists in a delicate balance that if perturbed can lead to mis-regulation of cell identity with potential pathogenic roles in myriad diseases, including cancer and renal fibrosis.

From a signalling perspective, EMT can be triggered by a number of different growth factors, prominent among which are the members of the Transforming growth factor (TGF)-β superfamily [9]. Intriguingly, this superfamily controls the fate of epithelial cells, with some of the cytokines belonging to the Bone Morphogenic Protein (BMP) family, in particular BMP-7, able to block and reverse EMT during renal injury [10]. TGFβ is a well-recognized player in the progression of renal disease [11] stimulating mesangial hypertrophy and deposition of Extracellular Matrix (ECM) proteins such as collagen and fibronectin [12]. It acts mainly though the activation of Smad2 and Smad3 transcription factors [13], which represent the “canonical” pathway and through the activation of a “non-canonical” signal pathway comprising mitogen-activated protein kinases (MAPK), Rho-like GTPases, and PI3K/Akt signalling [14].

It has been recently acknowledged that EMT is not, as initially believed, an “all-or-nothing” response, but it appears to be a delicately fine-tuned process characterized by intermediate “hybrid” phenotypes that are able to easily switch between the epithelial and mesenchymal states [15,16,17]. These cells remain poised in a partial EMT, suggesting an array of intermediate stages exist prior to phenotypic conversion.

This review will explore current and emerging transcriptional control mechanisms in the regulation of epithelial to mesenchymal transition and cellular plasticity in the kidney and examine parallel processes controlling the acquisition of pluripotency during iPSC generation. A more comprehensive understanding of these processes will shed light on the regulatory pathways that govern cell identity and identify new therapeutic paradigms for the treatment and regression of renal disease.

## 2. The Kidney at a Glance

The kidney is a highly specialized and complex organ composed of up to 30 different types of cells [18]. The basic structure of the kidney is comprised of nephrons, which represent the renal filtration units, and by the collecting duct system, which connects nephrons to the ureter [19]. The kidney has the peculiar ability to regenerate and restore its functionality after a certain degree of injury [20]. The mechanisms underlying renal regenerative capacity is still a matter of debate. A theory proposed by the group of Humphrey claim that restoration of tissue integrity is accomplished mostly through the dedifferentiation and proliferation of surviving epithelial cells [21,22,23]. Others have suggested the presence of resident renal progenitor cells that contribute to the renal regeneration after an injury [24,25]. These progenitor cells are CD133^+^ and CD24^+^ and are mostly localized within the Bowman’s capsule and all along the tubules [26], representing less than 2% of the total renal cells [27]. Regardless of the actual population of cells that carry out the repair process, the regenerative potential of the kidney has been demonstrated to be active only following an acute injury. This capacity is however impaired during chronic disease, due to prolonged insults that the kidney is unable to successfully resolve, leading eventually to End-Stage Renal Disease (ESRD) [28].

### Renal Development

Nephrogenesis is a complex and delicate process that leads to the formation of the nephrons. This process is completed between two and four postnatal days (PD) in the mouse, while in humans, is completed during the gestation phase [19]. The complete organ, the metanephric kidney, arises from two excretory organs that form during the gestational phase: the pronephric and the mesonephric kidney. These temporary organs derive from the intermediate mesoderm, which will form two fundamental structures during embryonic development: the Metanephric Mesenchyme (MM) and the Ureteric Bud (UB). In his pioneering work, Clifford Grobstein demonstrated that during kidney development the interaction between the MM and the UB was fundamental in order to achieve normal tubulogenesis. The sole interaction between these two cell compartments, rather than an actual contact, was required to successfully form the renal nephric epithelium [29]. The process through which mesenchymal cells of the MM give rise to the epithelial cells composing the nephron is a Mesenchymal to Epithelial Transition (MET) [30]. Before this event, however, the blastema is derived from primary epithelial cells through a reverse process of EMT; the mesoendoderm can thus be said to be generated by epiblasts [31]. In turn, the MM causes the UB to elongate and branch by secreting GDNF (Glial-cell line Derived Neurotrophic Factor). These invading branches induce the loose mesenchymal cells that surround the tips of the UB (cap mesenchyma) to form epithelial aggregates [19]. Many transcription factors are involved in regulating the process of nephrogenesis, including Six2, Pax2, and WT1. These factors are expressed in the nephron progenitor cells and their loss causes impairment in normal renal development due to the loss of the progenitor compartment [32,33,34,35]. Interestingly, Pax2 has been associated with EMT during the renal regenerative process. Pax2 is transiently upregulated six hours post folic acid injection in a model of renal failure in proximal tubular cells along with vimentin expression, a marker of mesenchymal cells [36]. Moreover, its re-expression protects the collecting duct cells from apoptosis in the unilateral ureteral obstructed (UUO) mouse, a widely accepted model of renal fibrosis [37]. Pax2 is downregulated after the reconstitution of the renal tubules, with an expression pattern similar to renal development. Intriguingly, WT1 is required to activate a program of EMT that sustains the formation of cardiac progenitor cells by inhibiting the expression of E-Cadherin and activating the expression of SNAI1/2 [38].

## 3. The Regenerative Potential of EMT

### 3.1. The Origin of the Myofibroblasts during Renal Disease

In the context of renal injury, EMT has historically been regarded as a negative and deleterious process and the main cause of fibrosis. It is widely accepted and proven that renal epithelial cells (and epithelial cells in general) *in vitro* are capable of undergoing an Epithelial to Mesenchymal Transition as a consequence of TGFβ stimulation [39,40,41]. This transition causes *de novo* expression of α-Smooth Muscle Actin (SMA) and production of ECM proteins, such as collagen type I, initiating a program of fibrosis [42]. The accumulated evidence led the scientific community to believe that the same process would take place *in vivo* and that the renal tubular epithelial cells were the major source of interstitial myofibroblasts. In particular, the crucial study supporting this theory is a cell fate tracing study published in 2002 by the group of Neilson [43]. The authors of this paper used bone marrow chimeras and transgenic reporter mice claiming that myofibroblasts arising in the kidney during fibrosis were mostly due to an EMT of the resident renal tubular epithelial cells. Their conclusions were supported principally by the results obtained by using transgenic mice in which the Green Fluorescent Protein (GFP) was under the control of the FSP-1 (Fibroblast Specific Protein-1) promoter, a protein that was believed to be specifically expressed by fibroblasts [44,45]. Recently, different groups have challenged the specificity of this marker as a pure fibroblast marker [46,47] and its reliability in identifying fibroblasts has been questioned [48]. Various groups have demonstrated that FSP-1^+^ cells express markers of mononuclear cells [48,49], invalidating its use as a proof of EMT-derived fibroblasts during renal failure. Moreover, recently LeBleu *et al.*, used a comprehensive fate tracking technology and reported that only around 5% of myofibroblasts can be accounted for by EMT and at least 50% are derived from resident fibroblasts [50].

Although several studies published in the last few years have questioned the actual contribution of renal epithelial cells to the generation of myofibroblasts [51,52,53] and a growing body of evidence supports the theory against tubular EMT as a primary player in renal fibrogenesis [50], the debate is still open [54] and it has been extensively reviewed and discussed elsewhere [55,56]. While this review was being written, a paper from the group of Nieto [57] confirmed the results obtained by LeBleu *et al.*, demonstrating that less than 1% of the renal tubular cells are responsible for the generation of interstitial myofibroblasts. They employed a transgenic mouse model in which the Tomato Fluorescent Protein was expressed under the control of the Kidney Specific Protein (KSP or Cadherin-16). This cadherin is expressed in the renal parenchyma exclusively in the epithelial cells of both the renal medulla and the cortex, so its expression is restricted to the epithelial compartment of the kidney. Furthermore, they found that epithelial cells in the renal tubules underwent a partial EMT, acquiring a hybrid phenotype during renal injury, in which they upregulated SNAI1 expression while maintaining their epithelial hallmarks. They also generated a transgenic mouse model in which the SNAI1 gene was deleted in KSP^+^ cells. They found that upon SNAI1 deletion in the epithelial cells of the kidney, mice were less prone to develop fibrosis compared to wild-type mice. According to the authors, by blocking the partial EMT of the renal epithelial cells they were able to prevent the release of inflammatory and fibrotic cytokines, such as TGFβ, in the microenvironment of the injured kidney and so preventing the generation of myofibroblasts and the development of fibrosis.

Nevertheless, what has clearly emerged from this debate is that renal tubular cells possess a high degree of plasticity, allowing them to adapt and respond to environmental stimuli.

### 3.2. Cellular Plasticity during Renal Repair

**Figure 2:**
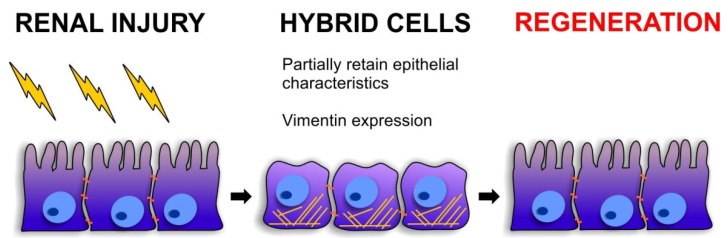
The role of intermediate “hybrid” cells during kidney regeneration. Following acute injury, renal epithelial cells are able to repair and recover their functionality. During this process, the renal epithelial cells undergo a process of profound plasticity in which they acquire a less epithelial phenotype and gain some mesenchymal characteristic, such as vimentin expression. These partially “reprogrammed” cells are able to proliferate and repair the damage.

As discussed previously, the kidney is able to regenerate and restore its function after an insult. It is now widely accepted that renal tubular epithelial cells respond, at least in part, to injury by de-differentiating into mesenchymal cells thus recapitulating the processes active during early nephrogenesis [58] and EMT functions in adults to facilitate tissue regeneration and regrowth during wound repair (Figure 2). Recently, Jiang *et al.*, have advanced the hypothesis that in the context of renal injury, EMT may be an event of phenotypic transition which reverses the process of tubular formation during embryonic development [59]. It is generally well accepted that renal epithelial cells, from both the nephron and the glomerulus, re-express markers typical of renal progenitors during the process of EMT and during nephrogenic disease. Glomerular parietal epithelial cells (GPECs) have been shown to spontaneously revert, *in vitro*, into an embryonic phenotype. In a study from 2011, Swetha *et al.*, demonstrated that cultured GPECs spontaneously undergo a process of EMT, acquiring expression of early renal progenitor markers [60]. These “progenitor-like cells” were successfully able to integrate into developing E13.5 kidneys in an *in vitro* system and gave rise to tubular structures once injected under the renal capsule in a model of unilateral nephrectomy, thus demonstrating that EMT confers plasticity to GPECs.

Interestingly two studies from the same group showed evidence of re-expression of two genes implicated in the initial stages of nephrogenesis, WT1 and Pax2 [61,62], in renal parenchymal undergoing EMT as a consequence of kidney injury. The authors outlined for the first time the possible involvement of the two genes in promoting EMT during renal regeneration and repair. Furthermore a recent paper by Hendry *et al.*, unraveled an unexpected role for the EMT-Transcription Factor (TF) SNAI2 in facilitating the direct reprogramming of adult cells into nephron progenitors [63]. Although this is the first report showing a putative role for SNAI2 in kidney development, this EMT-TF has already been demonstrated to play a pivotal role in renal branching [64]. SNAI1 and SNAI2 are two of the most important transcription factors that regulate EMT and they have also been implicated in cancer progression. Intriguingly, SNAI1 is required for tumor invasiveness but its continuous expression inhibits metastasis [65]. This interesting observation supports the theory that the most plastic and responsive cells are those that live in an intermediate phase, the “hybrid cells”. This is mostly accepted in the metastatic process of cancers, while fewer studies have been performed in respect to renal regeneration. Of note, the fundamental role of this partial EMT in kidney regeneration had been already recognized in 2007 by the work of Leroy and Mostov [64], who identified the involvement of “hybrid cells”, characterized by the presence of both mesenchymal (SNAI1 expression) and epithelial markers (E-Cadherin expression), in driving the branching of Madin-Darby Canine Kidney (MDCK) cells *in vitro*.

We have recently demonstrated that pluripotent stem cells isolated from the testis can differentiate into renal progenitor cells and successfully protect against renal failure [66]. These differentiated tubular-like cells stably express vimentin, along with E-Cadherin, like immature renal progenitor cells and are able to successfully protect the renal parenchyma against renal failure, whereas more differentiated cells (Vimentin^−^) are not able to restore renal functionality. Vimentin, not expressed in adult renal tubular cells, is highly upregulated in the first days after severe injury, (Figure 2). Intriguingly, during this process, renal tubular cells recapitulate gene expression patterns typical of the developing nephron. This reinforces the concept that EMT is needed in order for the cells to acquire a progenitor phenotype and might play a pivotal role in kidney regeneration. Moreover, it confirms that cells in an intermediate state have the greatest potential to participate in the repair process.

## 4. EMT and Stemness

The 2006 report by Takahashi and Yamanaka [6] represents a pivotal moment in regenerative medicine. In their paper they describe how murine embryonic fibroblasts were reprogrammed to become iPSCs by forced overexpression of various transcription factors. Since then new strategies and techniques have been developed and it is widely acknowledged that iPSC technology offers an unprecedented opportunity to obtain pluripotent stem cells free from ethical concerns linked to the use of human Embryonic Stem Cells (hESCs). One of the most important advantages of iPSCs is that they can be derived from patients suffering from genetically defined diseases, enabling investigators to better understand and characterize specific phenotypes caused by the diseases [67,68,69] and to use them for drug screenings [70] (reviewed in [71]). Furthermore, iPSC technology may enable us to gain new insight into cell fate determination. Unfortunately, the exact mechanism through which the cells acquire pluripotency is still relatively poorly understood and considered a stochastic process [72]. Detailed descriptions of the methodologies of iPSC generation and their application in regenerative medicine can be found in these reviews [73,74,75,76].

**Figure 3:**
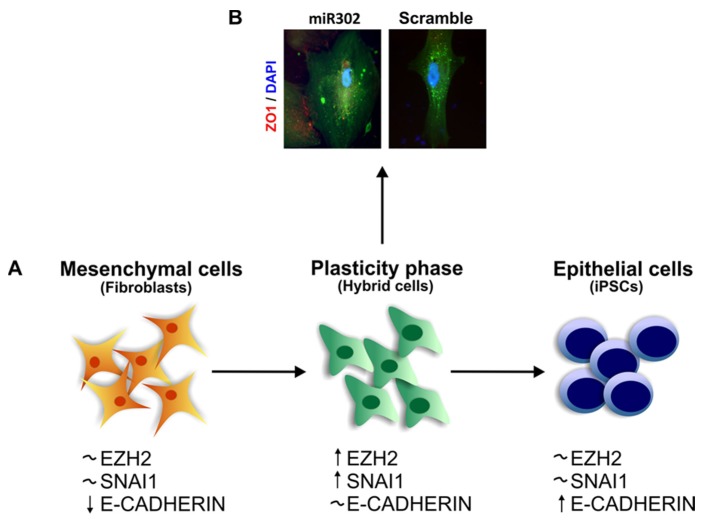
From mesenchymal to epithelial fate: routes to plasticity. Mesenchymal cells are characterized by a fibroblastic spindle-like phenotype. These cells do not express E-Cadherin and they have a low but detectable level of SNAI1 and EZH2. During transition to plasticity caused by forced expression of the 4 Yamanaka factors, these cells undergo a process of profound remodeling and they acquire the expression of both EZH2 and SNAI1. Once they become highly plastic and responsive, at a relatively low ratio, they acquire a fully pluripotent state (**A**). It is interesting to note that a similar process take places when human mesangial cells are forced to acquire a more plastic phenotype. While this process has something in common with fibrosis, the cells do not become fibrotic but they only acquire an enhanced plasticity. At this critical tipping point if these cells are grown in the right environment and with appropriate stimuli, they acquire epithelial characteristics and *de novo* expression of Zonula Occludens (ZO)-1 (**B**). This process is very dynamic and can be blocked or reversed.

Upon forced expression of reprogramming factors, the terminally differentiated cells pass through a phase of profound remodeling, characterized by the acquisition of plasticity, to eventually acquire a pluripotent state (Figure 3). As briefly discussed before, this route is a stochastic process and few cells are able to successfully reach their “final destination”, ~0.2% of the starting population in the case of human dermal fibroblasts [77]. It is interesting to note that EMT, and its reverse process MET, plays a pivotal role in directing the fate of iPSCs. Initially, the first papers that were published subsequent to the Yamanaka study, recognized only the importance of the MET during pluripotency acquisition [5]. It is not surprising that researchers focused their attention on MET without immediately acknowledging the importance of EMT. In fact, fibroblasts are a cellular type of mesenchymal origin, while the iPSCs are cells of epithelial nature, and so it would have been difficult to pinpoint a role for the opposite process, the EMT. One of the first reports that recognized the fundamental role of EMT during the acquisition of pluripotency is a 2013 paper by the group of Zheng *et al.* [7]. They demonstrated that the process of reprogramming can be improved by sequential introduction of the four original transcription factors, finding that the best combination is achieved by the addition of Oct4 and Klf4 first and then c-Myc followed by Sox2. Surprisingly, they showed that Oct4 induces the upregulation of SNAI2, causing a transient EMT and Oct4 has no effect on E-Cadherin expression. Moreover, they demonstrated that the same rate of iPSC generation can be obtained with the original protocol (all the four factors together) upon pre-treatment of the cells for 1.5 days with TGFβ to induce EMT. It is interesting to note that the best protocol of infection is achieved by the initial addition of Oct4 and Klf4 together, where Oct4 directly induces SNAI2 and Klf4 induces E-Cadherin, but not by Oct4 on its own. It is reasonable to infer that the explanation may reside in the fact that by adding these two transcription factors at the same time, the cells acquired an intermediate and more plastic phenotype that boosted them toward the acquisition of pluripotency.

### The Role of SNAI1 during Pluripotency and Plasticity Acquisition

The miR302 family of microRNA’s is composed of five members, miR302a/b/c/d and miR367. This family of microRNAs is expressed primarily in pluripotent cells and its expression is regulated by Oct4 and Sox2 by directly binding to the miR302 promoter [78,79]. It has recently been shown that iPSC generation can be promoted by overexpressing this cluster [80,81,82] (refer to this review for an in-depth analysis [83]). Our group have recently proved that miR302 expression is increased in mesangial cells undergoing growth factor mediated acquisition of plasticity [84] and that this process involves the regulation of the expression of SNAI1 [85].

This finding is not surprising since it is has been shown that overexpression of SNAI1 causes miR302 upregulation in murine ESCs [86], so there appears to be a clear reciprocal relationship. In addition, a challenging paper has recently demonstrated that SNAI1, and indeed EMT, is required to accomplish the acquisition of pluripotency [87]. Although it was previously reported that SNAI1 was up-regulated during the initial stage of the acquisition of pluripotency [88], this is the first paper that has tried to explain this paradox. The authors proved that SNAI1 is required not only to enhance but to successfully generate iPSCs. They showed that SNAI1 is capable of binding the promoter of let-7 family microRNAs impairing their expression, an intriguing observation given the relationship between let-7 and miR302 in regulating pluripotency [89,90]. Moreover they demonstrated, for the first time, that EMT occurs during the acquisition of pluripotency not only in mesenchymal cells but also in human keratinocytes, an epithelial cell type, suggesting that mesenchymal factors are an important aspect of reprogramming independent of the starting cell type. Keratinocytes, along with others type of epithelial cells, can be reprogrammed faster and with higher success rates compared to non-epithelial cell types [91,92]. This observation may be linked to the fact that by expressing SNAI1, the keratinocytes acquire an intermediate phenotype that makes them more plastic and easier to be reprogrammed. Unfortunately the authors do not specify the pattern of expression of E-Cadherin during ectopic overexpression of SNAI1 and so we can only speculate about the “hybrid” nature of these cells. Intriguingly, as noted in the case of metastatic cells, the temporary upregulation of SNAI1 promotes reprogramming, while its continuous expression inhibits the acquisition of pluripotency [5]. Furthermore, another paper from 2014 revealed an unexpected role for SNAI1 during the acquisition of pluripotency [93]. The authors used an initial approach of genome-wide RNAi to identify factors involved in promoting the final stage of the acquisition of pluripotency and they outlined a role for SNAI1 in that context. In particular they demonstrated how SNAI1 is required to modulate Nanog-driven pluripotency, although the authors reported that the role played by SNAI1 in that context is not correlated with its role in EMT. Notably, we found that human primary mesangial cells are able to acquire an epithelial phenotype after having gone through a phase of high plasticity characterized by the expression of SNAI1 (Figure 3) [85].

## 5. Chromatin Remodeling during EMT

### 5.1. The PRC2 Axis

As widely discussed in the previous paragraphs, EMT is a highly plastic process that involves genetic reprogramming and chromatin modifications that allow an epithelial cell to became a mesenchymal cell and vice versa. There are two major kind of chromatin modifications; a first layer of regulation is represented by DNA methylation, while a second layer is represented by the post translational modification of nucleosomal histone proteins in chromatin which play a critical role in the regulation of gene expression [94]; the latter is the focus of this section. Histone modifications can either activate or repress the expression of genes and they function as docking sites for chromatin modifiers that specifically recognize these modifications [95] and, in turn, recruit additional chromatin remodeling enzymes. Numerous ChIP-seq studies have demonstrated that cell type specific regulatory genes can be identified by the presence of specific histone marks [96,97]. Intriguingly, methylation of H3K4 and H3K26 are generally associated with active transcription [98] whereas permissive bivalent promoters in developmental genes are enriched with both active (H3K4me3) and repressive marks (H3K27me3) and are considered to exist in a “poised” state [99,100]. Central to these processes is the Polycomb Repressive Complex 2 (PRC2), the chromatin-modifying enzyme complex that methylates the histone H3 on lysine 27 [101]. The PRC2 complex is composed of three main subunits, SUZ12, EED and EZH2; EZH2 in particular (Enhancer of Zeste Homolog 2) plays a critical role in the complex due to its methyltransferase activity. Recently, it has been demonstrated that EZH2 also has a role as activator of transcription which is independent from its role in the PRC2 complex [102]. It is widely accepted that EZH2 overexpression is correlated with aggressiveness and invasive capacity in various types of cancers (see [103] for review) and it has been implicated in repressing the expression of E-Cadherin in nasopharyngeal cancer [104], renal cell carcinoma [105], oral tongue squamous carcinoma [106], and prostate and breast cancers [107]. Interestingly, SNAI2 cooperates with PRC2 to suppress the expression of E-Cadherin in cancer cells [104,108] and during embryonic development [109].

We have shown that the treatment of renal cells with a specific EZH2 inhibitor, blocks the TGFβ mediated epithelial dedifferentiation and the expression of genes associated with fibrosis. Furthermore, we noticed that upon inhibition of EZH2 methyltransferase activity, renal epithelial cells demonstrate an even higher level of E-Cadherin expression [85]. Of note, the same pattern of expression has been found in ESCs null for SUZ12 [108] and in cells in which EED has been knocked down [110].

Expression of EZH2 is characteristic of progenitor cells while its expression goes down during differentiation in various types of tissue [111,112]. The same pattern is found during renal development, in which EZH2 is expressed in renal progenitor cells [113] and cap mesenchyma [114] while it goes down during nephrogenesis. Renal progenitor cells have been characterized as cells with a mixed phenotype, expressing both vimentin and epithelial markers, but with a predominant plastic and mesenchymal behavior [115]. Interestingly, EZH2 is highly expressed by these renal progenitor cells along with Six2 and WT1, while it is repressed during differentiation and commitment [115]. Furthermore, a recent publication by the group of Rampalli has highlighted the role played by EZH2 mediated H3K27me3 activity during reprogramming [116].

The failure of EZH2 to be downregulated has been proposed as one of the causes of Wilms tumor phenotype and behavior [117]. In this type of aggressive renal tumor, the cells fail to undergo differentiation and retain a progenitor phenotype as a consequence of sustained EZH2 expression. Furthermore, the PRC2 components have been shown to be upregulated during EMT and involved in the EMT progression [110,118].

### 5.2. The S”MAD” Idea

Genome-wide profiling of histone methylation during EMT revealed strong correlations between the dynamic changes of histone methylation and gene expression [119]. Intriguingly, a paper published in 2015 demonstrated that during EMT, SNAI1/2 bind and repress the promoter of miR101 causing the upregulation of EZH2 facilitating the invasion of oral tongue carcinoma and the generation of stem cell-like features [120], providing an interesting link between the EMT master transcription factor SNAI1 and EZH2 expression during EMT. Activation of SNAI1 during EMT is driven, among other different molecules, by TGFβ stimulation [121]. TGFβ initiates canonical and non-canonical pathways to exert multiple biological effects. These processes include stem cell maintenance [122], cell proliferation [123], differentiation [122], apoptosis [124], and embryonic development [125]. Moreover, TGFβ is one of the most important proteins involved in EMT [9]. The activation of its receptor leads to the phosphorylation of two transcription factors that belong to the Smad (Mothers Against Decapentaplegic) family, Smad2 and Smad3. Once activated, Smad2/3 translocate into the nucleus where they can interact with DNA, either directly (Smad3) through a SMAD Binding Element (SBE) or indirectly (Smad2 and Smad3) by associating with Smad4. In 2011 Mullen and colleagues [126] found that during the acquisition of pluripotency and direct trans-differentiation, master transcription factors co-occupy the genome with Smad2/3 and thus are responsible for directing the gene targets of TGFβ signaling, ultimately determining its cell-specific effects. A recent study by the group of Kaji demonstrated that upon forced expression of a constitutively active form of Smad2/3, cells can be reprogrammed more efficiently. Intriguingly, they showed also that Smad3 can interact with a multitude of nucleosome remodeling complexes, including p300, Dpy30 and negative repressive complexes [127]. They hypothesized that, Oct4, a master transcription factor of pluripotency forms a complex with SMAD3 and in this way it recruits transcriptional activator complexes. It is interesting to note that we have evidence that this “recruitment” function is exerted in the same way by Smad3 with transcriptional repressive complexes [128].

## 6. Conclusions

**Figure 4:**
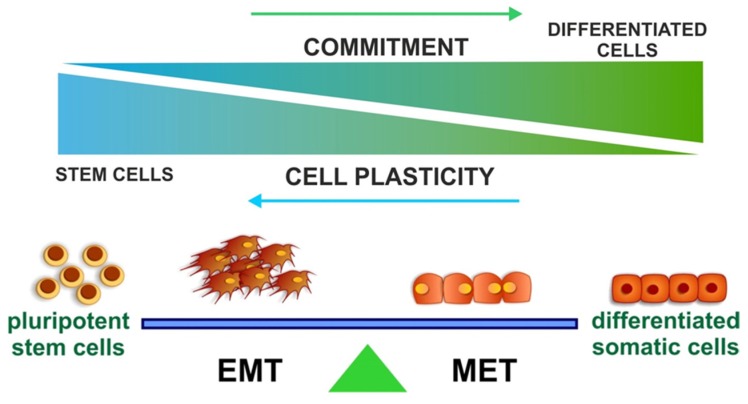
Working model. The balance between Epithelial and Mesenchymal phenotype controls various processes during embryogenesis, development, diseases, and acquisition of pluripotency. By better understanding how this process is tuned-in and regulated, we may be able to stop the progression and initiate the regression of multiple fibrotic disease, such as the one affecting the kidney.

In the 20 years since Frank Strutz *et al.*, first identified the presence of FSP-1 positive cells within renal tubules and proposed Epithelial to Mesenchymal Transitions as a theory of renal fibrosis, significant advances have been made in the fundamental biology underlying cell fate in disease. Advances in cell fate tracing technology first questioned this hypothesis and more recently have defined the origin of myofibroblasts within the diseased kidney; it appears that EMT can only account for a small number of these cells. Nevertheless, transitions between epithelial and mesenchymal phenotypes are linked to the acquisition of a stem cell-like “plastic” stage and recent findings suggest that this plasticity may be the key to therapies that hold the promise of fibrotic regression. Whereas cells require EMT to acquire features of stem cells, an MET is needed to successfully reprogram cells to a pluripotent stage. It seems therefore reasonable to hypothesise that highly plastic cells, possessing an intermediate “hybrid” phenotype, can be shifted to the epithelial or mesenchymal fate (Figure 3) by the differentiation microenvironment. A plastic cell within a modified microenvironment and in the right context may aid resolution of injury and restore organ functionality. The corollary is that the same cell stimulated by a fibrotic and pro-inflammatory environment may lead the organ to end-stage disease (Figure 4). The next steps require a more fundamental understanding of the interplay between transcriptional regulatory networks and extracellular cues, in particular the dissection of the epigenetic mechanism controlling dynamic repression and activation of gene expression. Obvious parallels exist between induced pluripotency, organogenesis/fate determination, and disease and are potentially exploitable for therapeutic gain.
